# Interaction between *FTO* rs9939609 and the Native American-origin *ABCA1* rs9282541 affects BMI in the admixed Mexican population

**DOI:** 10.1186/s12881-017-0410-y

**Published:** 2017-05-02

**Authors:** Marisela Villalobos-Comparán, Bárbara Antuna-Puente, María Teresa Villarreal-Molina, Samuel Canizales-Quinteros, Rafael Velázquez-Cruz, Paola León-Mimila, Hugo Villamil-Ramírez, Juan Antonio González-Barrios, José Luis Merino-García, María Rocío Thompson-Bonilla, Diego Jarquin, Osvaldo Erik Sánchez-Hernández, Martha Eunice Rodríguez-Arellano, Carlos Posadas-Romero, Gilberto Vargas-Alarcón, Francisco Campos-Pérez, Manuel Quiterio, Jorge Salmerón-Castro, Alessandra Carnevale, Sandra Romero-Hidalgo

**Affiliations:** 1 Consorcio Genómica Computacional, Instituto Nacional de Medicina Genómica, Mexico City, Mexico; 2 Laboratorio de Genómica de Enfermedades Cardiovasculares, Instituto Nacional de Medicina Genómica, Mexico City, Mexico; 3 Unidad de Genómica de Poblaciones Aplicada a la Salud, Facultad de Química, UNAM-INMEGEN, Mexico City, Mexico; 4 Laboratorio de Genómica del Metabolismo Óseo, Instituto Nacional de Medicina Genómica, Mexico City, Mexico; 5 Hospital Regional “Primero de Octubre”, Instituto de Seguridad y Servicios Sociales de los Trabajadores del Estado, Mexico City, Mexico; 6 Department of Agronomy and Horticulture, University of Nebraska, Lincoln, NE USA; 7 Hospital Regional “Lic. Adolfo López Mateos”, Instituto de Seguridad y Servicios Sociales de los Trabajadores del Estado, Mexico City, Mexico; 8 Departamento de Endocrinología, Instituto Nacional de Cardiología “Ignacio Chávez”, Mexico City, Mexico; 9 Departamento de Biología Molecular, Instituto Nacional de Cardiologia “Ignacio Chávez”, Mexico City, Mexico; 10 Clínica Integral de Cirugía para la Obesidad y Enfermedades Metabólicas, Hospital Rubén Leñero, Mexico City, Mexico; 11 Centro de Investigación en Salud Poblacional, Instituto Nacional de Salud Pública, Cuernavaca, Morelos Mexico; 12 Unidad de Investigación Epidemiológica y en Servicios de Salud, Instituto Mexicano del Seguro Social, Cuernavaca, Morelos Mexico; 13 Laboratorio de Enfermedades Mendelianas, Instituto Nacional de Medicina Genómica, Mexico City, Mexico

**Keywords:** Body mass index, *FTO* and *ABCA1* variants, Interaction

## Abstract

**Background:**

The aim of this study was to explore whether interactions between *FTO* rs9939609 and *ABCA1* rs9282541 affect BMI and waist circumference (WC), and could explain previously reported population differences in *FTO*-obesity and *FTO*-BMI associations in the Mexican and European populations.

**Methods:**

A total of 3938 adults and 636 school-aged children from Central Mexico were genotyped for both polymorphisms. Subcutaneous and visceral adipose tissue biopsies from 22 class III obesity patients were analyzed for *FTO* and *ABCA1* mRNA expression. Generalized linear models were used to test for associations and gene-gene interactions affecting BMI, WC and FTO expression.

**Results:**

*FTO* and *ABCA1* risk alleles were not individually associated with higher BMI or WC. However, in the absence of the *ABCA1* risk allele, the FTO risk variant was significantly associated with higher BMI (*P* = 0.043) and marginally associated with higher WC (*P* = 0.067), as reported in Europeans. The gene-gene interaction affecting BMI and WC was statistically significant only in adults. *FTO* mRNA expression in subcutaneous abdominal adipose tissue according to *ABCA1* genotype was consistent with these findings.

**Conclusions:**

This is the first report showing evidence of *FTO* and *ABCA1* gene variant interactions affecting BMI, which may explain previously reported population differences. Further studies are needed to confirm this interaction.

**Electronic supplementary material:**

The online version of this article (doi:10.1186/s12881-017-0410-y) contains supplementary material, which is available to authorized users.

## Background

The *FTO* rs9939609 gene variant has been consistently associated with BMI and obesity, however clear population differences have been identified [1]. Despite the high prevalence of obesity in Mexico, the *FTO* risk allele is considerably less frequent, both in admixed and Native populations as compared to Europeans (0.21, 0.06 and 0.46, respectively). Interestingly, rs9939609 has been associated only with class III obesity, but not with overall obesity or BMI in admixed Mexican adults [2, 3], and rs1421085, in high linkage disequilibrium with rs9939609, was not associated with obesity or BMI in admixed Mexican children [4]. It has been stated that loci that are specific to a single ancestry might contribute to genetic susceptibility across populations [5]. The *ABCA1-*R230C variant (rs9282541) is an ancestry-specific polymorphism private to the Americas and has been strongly associated with low HDL-cholesterol (HDL-C), although its association with BMI and obesity is inconsistent [6–8]. This allele is of particular interest, because it is relatively frequent in the Mexican mestizo population (0.11), is functional and was found to interact with BMI affecting abdominal fat distribution particularly in Mexican premenopausal women [8]. The aim of this study was to analyze possible *FTO* rs9939609 - *ABCA1* rs9282541 interactions affecting BMI, waist circumference (WC) and HDL-C levels in individuals from Central Mexico, which could help explain the differences observed between this and European populations.

## Methods

### Study population description

The studied population included 3938 DNA samples of unrelated Mexican mestizo adults from 4 different cohorts and 636 DNA samples of unrelated school-aged children. All cohorts include samples from Central Mexico and have been previously described (Table 1). Protocols for each cohort were approved by their respective Institutional Ethics Committee. Fully informed written consent for participation was attained from all participants or legal guardians.

**Table 1:** Description of the study cohorts

Study (Reference)	Sample size (% ancestry^a^)	Region (State)	Males (%)	Mean age (years ± SD)	Mean BMI (Kg/m^2^ ± SD)	Mean WC (cm ± SD)	Mean HDL-C (mg/dL ± SD)
Romero-Hidalgo et al. [16]	525 (67.5)	Central Mexico (Mexico City, Hidalgo, Edo. De México, Morelos, Querétaro)	31.2%	46.2 ± 13.6	27.7 ± 4.5	92.0 ± 14.1	44.8 ± 12.7
Velázquez-Cruz et al. [17]	1207 (50.6)	Central Mexico (Morelos)	30.3%	50.9 ± 15.3	27.0 ± 4.5	93.7 ± 10.8	44.3 ± 11.4
Villarreal-Molina et al. [8]	1511 (72.5)	Central Mexico (Mexico City)	50.9%	53.1 ± 9.3	28.4 ± 4.3	94.8 ± 11.6	45.9 ± 13.3
Villalobos-Comparán et al. [2]	695 (67.5)	Central Mexico (Mexico City)	36.0%	40.0 ± 13.6	27.2 ± 5.2	89.1 ± 13.4	46.3 ± 12.5
León-Mimila et al. [3]	636	Central Mexico (Mexico City)	48.23%	9.4 ± 1.85	20.0 ± 3.84	70.2 ± 11.3	46.8 ± 10.9

^a^Proportion of individuals with an ancestry estimation

### Genotyping

The *FTO* rs9939609 and *ABCA1* rs9282541 variants were genotyped in 3938 DNA samples using TaqMan assays (ABI Prism 7900HT Sequence Detection System, Applied Biosystems). In addition, because the Mexican-Mestizo population is admixed, individual ancestry estimates were analyzed for 2354 individuals to test whether the results could be confounded by population stratification. Different panels of ancestry informative markers were used for each cohort (Additional file 1: Table S1).

### Expression analysis

*FTO* and *ABCA1* mRNA expression was measured in subcutaneous (SAT) and visceral (VAT) adipose tissue biopsies from 22 admixed Mexican patients (16 female and 6 male), aged 25 to 55 years with BMI > =40 kg/m^2^, who underwent bariatric surgery at the Hospital General Rubén Leñero in Mexico City. Total RNA was extracted with RNeasy Lipid Tissue Mini Kit (Qiagen), cDNA was reverse transcribed with TaqMan Reverse Transcription Reagents Kit (Applied Biosystems). Expression was analyzed using GeneChip Human Genome 2.0 ST Array (Affymetrix). *FTO* and *ABCA1* expression were validated by Real-Time PCR (LightCycler 480 II, Roche), using the following primers and probes: ctcggagaattagtttaggatatttca (forward) tctgacccccaaagatgatg (reverse) and probe #59 for *FTO*, and tgctgcatagtcttgggactc (forward), acctcctgtcgcatgtcact (reverse) and probe #17 for *ABCA1*. Hypoxanthine phosphoribosyl transferase (*HPRT*) expression was measured as reference [2].

### Statistical methods

HDL-C measurements were log-transformed for the analysis. Generalized linear regression (GLM) models were used to evaluate the individual effect of each single nucleotide variant and the interaction between *FTO* and *ABCA1* risk variants, adjusting for age, gender, ancestry and BMI as appropriate. GLM models were also used to compare mean values of subcutaneous and visceral *FTO* gene expression, adjusted for age and gender. Thus a model with main effects for risk variants and the adjusted variables plus the interactions between risk variants was fitted. All statistical analyses were performed using SPSS v.15.

## Results

*FTO* and *ABCA1* risk allele frequencies were 20.3 and 10.0% in the overall population, respectively. In order to avoid potential population stratification, all individuals included in the analysis were from Central Mexico.

**Fig. 1:**
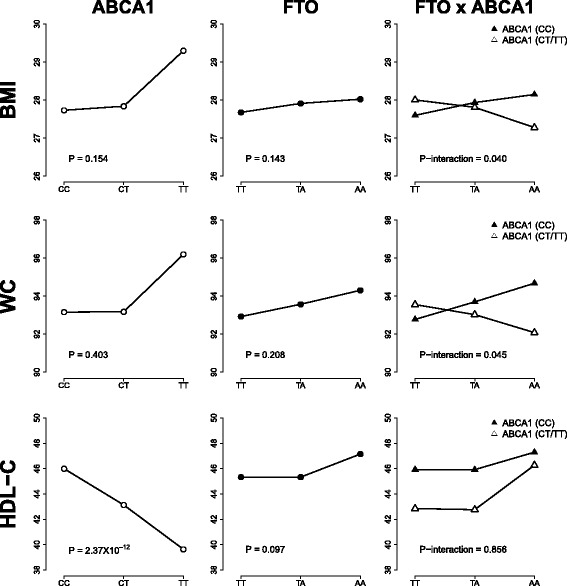
Association of *FTO* rs9939609 and *ABCA1* rs9282541 risk variants, independently and stratified, with BMI, waist circumference (WC) and HDL-cholesterol (HDL-C) levels, adjusting for age, gender and BMI as appropriate

In the overall analysis and under additive inheritance models, the *FTO* “A” risk allele was not significantly associated with higher BMI (β = 0.187, *P* = 0.143) or higher WC (β = 0.409, *P* = 0.208), nor was the *ABCA1* “T” risk allele associated with BMI or WC (β = 0.247, *P* = 0.154 and β = 0.369, *P* = 0.403, respectively). Furthermore, although HDL-C levels were higher in *FTO* “A” homozygous individuals, the association did not reach statistical significance (β = 0.005, *P* = 0.097). As expected, the *ABCA1* “T” allele was strongly associated with lower HDL-C levels (β = −0.03, *P* = 2.37 × 10^−12^) (Fig. 1).

In order to assess a possible *FTO-ABCA1* gene interaction, we sought associations between the *FTO* risk variant and BMI, WC and HDL-C, stratified by the absence or presence of the *ABCA1* risk allele (“CC” and “CT/TT” genotypes, respectively). Interestingly, in the absence of the *ABCA1* risk “T” allele, the *FTO* risk variant was significantly associated with higher BMI (β = 0.284, *P* = 0.042, *n* = 3191) and marginally associated with higher WC (β = 0.650, *P* = 0.063, *n* = 3191). In contrast, in the presence of the *ABCA1* risk allele, the *FTO* risk variant was not associated with BMI (*P* = 0.421, *n* = 747) or WC (*P* = 0.376, *n* = 747). The interaction analyses between *FTO* rs9939609 and *ABCA1* rs9282541 affecting BMI and WC were statistically significant (*P* = 0.040 and *P* = 0.045, respectively). *ABCA1* and *FTO* gene variants showed no significant interaction affecting HDL-C levels (*P* = 0.856) (Fig. 1).

**Fig. 2:**
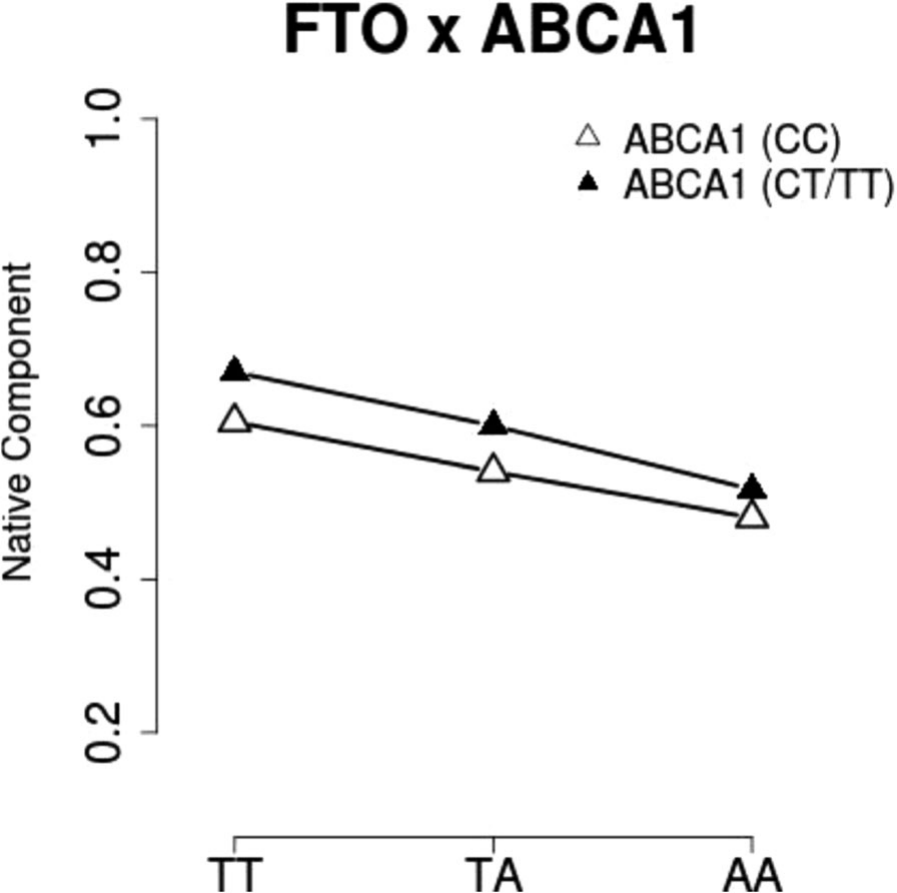
Mean Native American ancestry proportion according to FTO rs99399609 genotype, stratified by ABCA1 rs9282541 genotype in 2354 individuals

Individual ancestry estimates were available in 60% of the samples. After adjusting for Native American ancestry, the statistical significance of interactions between *FTO* rs9939609 and *ABCA1* rs9282541 affecting BMI and WC was borderline significant (*P* = 0.054 and *P* = 0.063, respectively). This drop of significance is probably due to the lower sample size. Figure 2 shows the mean proportion of Native American component according to *FTO* genotype, stratified by the absence or presence of the *ABCA1* risk allele (“CC” and “CT/TT” genotypes). As expected, the Native American component was lower in individuals with 1 and 2 FTO risk alleles, regardless of the presence of the ABCA1 risk allele. This suggests that the interactions are not confounded by ancestry.

We sought to evaluate this finding in an independent cohort of 636 children. As observed in adults, in the overall analysis using an additive inheritance model, the association of *FTO* “A” and the *ABCA1* “T” risk alleles with BMI percentile did not reach statistical significance (β = 3.116, *P* = 0.084 and β = 4.002, *P* = 0.058, respectively). However, in the absence of the *ABCA1* “T” risk variant, the effect of *FTO* risk allele over the BMI was higher and significant (β = 4.20, *P* = 0.043, *n* = 505), although the interaction did not reach significance (*P* = 0.356).

**Fig. 3:**
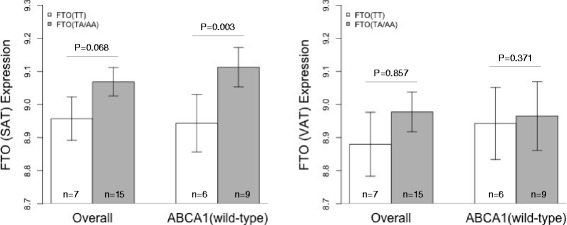
*FTO* mRNA expression levels in subcutaneous (SAT) and visceral adipose tissue (VAT) biopsies, in all tested biopsies (*n* = 22), and in individuals not bearing the *ABCA1* rs9282541 risk variant (wild-type, *n* = 15). *P*-values were obtained adjusting by age and gender

We further explored whether *FTO* mRNA expression is affected by *ABCA1* genotypes. Figure 3 shows differences in relative *FTO* mRNA expression levels in human adipose tissue biopsies according to *FTO* rs9939609 genotypes under a dominant model. In the overall analysis of SAT biopsies, while *FTO* mRNA expression was higher for “TA/AA” than those with “TT” genotypes, the difference did not reach statistical significance (9.084 vs 8.961 AU, respectively; *P* = 0.068). However, considering only biopsies of individuals not bearing the *ABCA1* risk allele (wild-type), *FTO* “TA/AA” SAT biopsies showed significantly higher *FTO* mRNA expression than those with “TT” genotypes (9.112 vs 8.943 AU, respectively; *P* = 0.003). Comparisons of *FTO* mRNA expression according to genotype in individuals bearing the *ABCA1* risk variant were limited because only one individual carried the *FTO* “TT” genotype. However, the *FTO* “TA/AA” SAT biopsies showed significantly lower *FTO* mRNA expression levels in the presence of the *ABCA*1 risk allele (9.043 vs 9.112, *P* = 0.045). In VAT biopsies, *FTO* mRNA expression did not differ significantly according to genotype in the overall population (TT: 8.879 vs TA/AA: 8.975, P = 0.857), or in absence of the R230C risk allele (TT: 8.942; TA/AA: 8.965, *P* = 0.371). *ABCA1* expression was not significantly affected by *FTO* rs9939609 genotypes (data not shown).

## Discussion

According to WHO, Mexico has one of the highest rates of adulthood and childhood obesity. This higher prevalence of obesity as compared to European populations could be explained by the Native American component as the result of adaptive processes related to energy saving, or could be the result ancestry-specific allele combinations derived from the admixture process. We thus analyzed whether the functional private *ABCA1*-R230C risk allele might interact with the most replicated obesity risk allele *FTO* rs9939609.

*FTO* and *ABCA1* risk allele frequencies were 20.3% y 10%, respectively, similar to previous reports [2, 3]. Individually, *FTO* and *ABCA1* risk alleles showed no significant association with BMI or WC. However, in the absence of the *ABCA1* risk variant, the effect of *FTO* on BMI and WC became stronger, statistically significant and similar to what has been reported in European populations [9]. Observations from the cohort of children showed a similar trend, although the gene-gene interaction reached statistical significance only in the adult cohort. The lack of significance in children was likely due to the small sample size. Replication studies in independent adult and childhood cohorts are necessary to confirm this interaction. This type of interaction may explain differences in *FTO* associations with obesity between Mexican and European populations, but they do not explain the higher prevalence of obesity in Mexico.

*FTO* mRNA expression was significantly higher in SAT than in VAT, in accordance with previous studies in other populations [10]. Interestingly, allele-specific *FTO* expression in SAT differed significantly only in the absence of the *ABCA1* risk allele, which is consistent with the interactions described above. It is noteworthy that no significant differences in allele-specific *FTO* expression have been observed in SAT biopsies from European individuals [11, 12]. However, a previous study in Mexican patients with morbid obesity, the rs9939609 “TA” genotype was significantly associated with higher *FTO* expression [2]. Although the latter biopsies were not genotyped for *ABCA1-*R230C, it is noteworthy that the only independent studies reporting allele-specific differences were performed in Mexican patients.

Although *FTO* and *ABCA1* are both known to play a role in adipose tissue function, there is no previous experimental evidence directly linking the function of both genes [13, 14]. However, previous evidence supports the role of *ABCA1* in body fat distribution both in the Mexican population [8], and in a recent multi-ethnic meta-analysis that identified *ABCA1* rs10991437 as a variant associated with higher waist-hip ratio adjusted for BMI [15].

## Conclusions

To our knowledge this is the first report showing evidence of FTO and ABCA1 gene variant interactions affecting BMI, which may explain previously reported population differences. Further studies are needed to understand the possible biological mechanisms underlying this interaction.

## Additional file

Additional file 1: Panels of ancestry informative markers used for each cohort [18]. (DOC 30 kb)

## Abbreviations

ABCA1: ATP Binding cassette Transporter A-1
BMI: Body mass index
FTO: Fat mass and obesity associated gene
HDL-C: High Density Lipoprotein Cholesterol
WC: Waist circumference
WHO: World Health Organization

## References

[1] Qi Q, Kilpeläinen TO, Downer MK, Tanaka T, Smith CE, Sluijs I (2014). FTO genetic variants, dietary intake and body mass index: insights from 177,330 individuals. Hum Mol Genet.

[2] Villalobos-Comparán M, Flores-Dorantes MT, Villarreal-Molina MT, Rodríguez-Cruz M, García-Ulloa AC, Robles L (2008). The *FTO* gene is associated with adulthood obesity in the Mexican population. Obesity.

[3] León-Mimila P, Villamil-Ramírez H, Villalobos-Comparán M, Villarreal-Molina T, Romero-Hidalgo S, López-Contreras B (2013). Impact of common genetic variants on childhood and adult obesity in the Mexican population. PLoS One.

[4] Mejía-Benítez A, Klünder-Klünder M, Yengo L, Meyre D, Aradillas C, Cruz E (2013). Analysis of the contribution of *FTO*, *NPC1*, *ENPP1*, *NEGR1*, *GNPDA2* and *MC4R* genes to obesity in Mexican children. BMC Med Genet.

[5] Lu Y, Loos RJ (2013). Obesity genomics: assessing the transferability of susceptibility loci across diverse populations. Genome Med.

[6] Villarreal-Molina T, Aguilar-Salinas CA, Rodríguez-Cruz M, Riaño D, Villalobos-Comparan M, Coral-Vazquez R (2007). The ATP-binding cassette transporter A1 R230C variant affects HDL cholesterol levels and BMI in the Mexican population: association with obesity and obesity-related comorbidities. Diabetes.

[7] Acuña-Alonzo V, Flores-Dorantes T, Kruit JK, Villarreal-Molina T, Arellano-Campos O, Hünemeier T (2010). A functional *ABCA*1 gene variant is associated with low HDL-cholesterol levels and shows evidence of positive selection in Native Americans. Hum Mol Genet.

[8] Villarreal-Molina T, Posadas-Romero C, Romero-Hidalgo S, Antúnez-Argüelles E, Bautista-Grande A, Vargas-Alarcón G (2012). The *ABCA*1 gene R230C variant is associated with decreased risk of premature coronary artery disease: the genetics of atherosclerotic disease (GEA) study. PLoS One.

[9] Tung YC, Yeo GS, O’Rahilly S, Coll AP (2014). Obesity and *FTO*: changing focus at a complex locus. Cell Metab.

[10] Klöting N, Schleinitz D, Ruschke K, Berndt J, Fasshauer M, Tönjes A (2008). Inverse relationship between obesity and *FTO* gene expression in visceral adipose tissue in humans. Diabetologia.

[11] Wåhlén K, Sjölin E, Hoffstedt J (2007). The common rs9939609 gene variant of the fat mass and obesity associated gene (*FTO*) is related to fat cell lipolysis. J Lipid Res.

[12] Zabena C, González-Sánchez JL, Martínez-Larrad MT, Torres-García A, Alvarez-Fernández-Represa J (2009). The FTO obesity gene. Genotyping and gene expression analysis in morbidly obese patients. Obes Surg.

[13] Zhang M, Zhang Y, Ma J, Guo F, Cao Q, Zhang Y (2015). The demethylase activity of FTO (Fat mass and obesity associated protein) is required for preadipocyte differentiation. PLoS One.

[14] De Haan W, Bhattacharjee A, Ruddle P, Kang MH, Hayden MR (2014). ABCA1 in adipocytes regulates adipose tissue lipid content, glucose tolerance, and insulin sensitivity. J Lipid Res.

[15] Shungin D, Winkler TW, Croteau-Chonka DC, Ferreira T, Locke AE, Mägi R (2015). New genetic loci link adipose and insulin biology to body fat distribution. Nature.

[16] Romero-Hidalgo S, Villarreal-Molina T, González-Barrios J, Canizales-Quinteros, Rodríguez-Arellano M, Yañez-Velazco LB (2012). Carbohydrate intake modulates the effect of the *ABCA1*-R230C variant on HDL cholesterol concentrations in premenopausal women. J Nutr.

[17] Velázquez-Cruz R, Jiménez-Ortega RF, Parra-Torres AY, Castillejos-López M, Patiño N, Quiterio M (2014). Analysis of association of *MEF2C*, *SOST* and *JAG1* genes with bone mineral density in Mexican-Mestizo postmenopausal women. BMC Musculoskelet Disord.

[18] Kosoy R, Nassir R, Tian C, White PA, Butler LM, et al. Ancestry informative marker sets for determining continental origin and admixture proportions in common populations in America. Hum Mutat. 2009;30(1):69–78. 10.1002/humu.20822 PMC3073397 18683858

