# Supplementary material for: Interaction between FTO rs9939609 and the Native American-origin ABCA1 rs9282541 affects BMI in the admixed Mexican population
Source: BMC Med Genet. 2017 May 2;18:46. doi: 10.1186/s12881-017-0410-y (PMC5414298; doi:10.1186/s12881-017-0410-y)
Supplement: Additional file 1: — Panels of ancestry informative markers used for each cohort [18]. (DOC 30 kb) [file 12881_2017_410_MOESM1_ESM.doc]

Additional file 1: Table S1

| **Study (Reference)** | **Number of AIM's** | **AIM's Description** |
| --- | --- | --- |
| Romero-Hidalgo *et al*. [16] | 18 | rs4908736, rs2225251, rs719776, rs1403454, rs2702414, rs26247, rs1881826, rs2396676, rs1373302, rs4130405, rs2149589, rs1487214, rs4924116, rs1014263, rs718387, rs5762401, rs878825, rs132628 |
| Velázquez-Cruz *et al*. [17] | 96 | Velázquez-Cruz *et al*. [17] |
| Villarreal-Molina *et al.* [8] | 95 | Kosoy et al. [18] |
| Villalobos-Comparán *et al*. [2] | 10 | Villalobos-Comparán *et al*. [2] |
